# The Influence of Family Milieu on Dental Anxiety in Adolescents—A Cross-Sectional Study

**DOI:** 10.3390/jcm12062174

**Published:** 2023-03-10

**Authors:** Nir Uziel, Joseph Meyerson, Moataz Kuskasy, Efrat Gilon, Ilana Eli

**Affiliations:** 1 Unit of Medical Education, Department of Oral Rehabilitation, The Maurice and Gabriela Goldschleger School of Dental Medicine, Sackler Faculty of Medicine, Tel Aviv University, Tel Aviv 6997801, Israel; 2 The Department of Master of Arts in Medical Psychology, The Max Stern Yezreel Valley College, Emeq Yezreel 1930600, Israel

**Keywords:** dental anxiety, dental fear, DAS, adolescents, memories, parents, family milieu, education

## Abstract

Parents play a significant role in the development of dental anxiety in their adolescent children. This cross-sectional study aimed to evaluate the influence of family milieu on adolescents’ dental anxiety. The level of dental anxiety (Dental Anxiety Scale—DAS) was evaluated in 100 adolescent dental patients (ages 13–18 years) and their parents. Parents supplied information about family demographics and their personal experiences of dental treatment. Concomitantly, the family’s behavior was observed during the dental encounter. The adolescents’ mean DAS score was 9.83 ± 2.05. Adolescents’ DAS was higher when their parents’ memories from their own dental encounters were negative. A positive correlation was found between adolescents’ dental anxiety and that of their father (r = 0.52, *p* < 0.001) and mother (r = 0.79, *p* < 0.001). The following variables showed a significant ability to predict adolescents’ dental anxiety (stepwise regression): mother’s DAS (B = 0.57), adolescent’s behavior at the dental visit (B = 0.87), being the firstborn child (B = −0.44), father’s DAS (B = 0.13), and mother’s level of education (B = −0.10). The results showed that intra-family relationships and behavior, parents’ education, dental fear, and memories from previous treatments play an important role in defining the level of dental anxiety in their adolescent children.

## 1. Introduction

Dental fear is a common problem. The estimates are that about 6–15% of the global population suffers from a high level of dental fear and avoidance [1].

Dental fear and anxiety are believed to be maintained by a vicious cycle. The presence of the vicious cycle was demonstrated by Armfield in a study on 1036 subjects. The study showed that dental fear acts as a determinant of avoiding or delaying dental visits, which has flow-on effects in terms of further treatment needed and problem-oriented visiting [2]. The avoidance of routine dental care leads to a higher incidence of pain which in turn affects anxiety and vice versa.

The treatments that cause the highest anxiety in dental care are usually those affiliated with different aspects of oral surgery [3]. In a situation involving periodontal surgery, a significant increase in the subject’s anxiety was observed immediately before surgery [4].

Furthermore, dental anxiety and pain during dental treatment are closely interrelated. For example, the pain experienced by patients in oral surgery was best predicted by their anxiety at each time point [5]. Therefore, the proper understanding and management of the patient’s anxiety are, in many cases, mandatory to reduce their subjective pain experience before, during, and following treatment.

The most well-known measure for assessing dental anxiety, the Dental Anxiety Scale (DAS), was developed by Corah [6]. The DAS is commonly used as a specific measure of dental anxiety [7,8,9,10]. It comprises four items concerning four dentally related scenarios, where respondents are asked to select the option closest to their probable response to each situation. Items are scored on a scale of 1 (no anxiety) to 5 (extreme anxiety) and summed to give an overall anxiety score ranging from 4 to 20. DAS can be used in adults, children, and adolescents [7,8,9,10].

Since dental anxiety has a complex and multifactorial etiology, studies of the reasons for the development of dental anxiety face numerous problems. Subjects who come from varied socio-economic and cultural backgrounds are exposed to different dental experiences during their childhood and early adolescence. Thus, the definition of the main cause of the development of the syndrome is complex.

Three main aspects have been suggested as affecting the development of dental anxiety: (i) direct conditioning, originating from early aversive encounters in the dental office [11,12,13]; (ii) vicarious learning, through family, peers, and society [13,14]; and (iii) psychodynamic and personality aspects, namely specific traits that, when present, increase the patient’s tendency to feel apprehension in the dental setting [7,15]. For example, Eli et al. [8] reported that the level of a subject’s dental anxiety is affected by both environmental factors and personality traits.

The family milieu can affect one’s emotions and behavior during dental care in many ways. Aside from vicarious learning, a mode of attachment has the potential to substantially affect one’s ability to cope with dental treatment. Attachment theory claims that parental responsiveness and availability to the infant in times of distress provide the child with a “secure base” upon which to organize experiences and handle stress [16]. The early experiences that shape the individual attachment pattern become an inner representation, which organizes cognition and affects behavior throughout life [16]. Three attachment styles were identified—secure, ambivalent, and avoidant [17]. It was shown that attachment pattern has a final effect on whether dental anxiety persists throughout life or can be modulated through a corrective emotional experience [18].

Dental anxiety is thought to originate in childhood [11], peak in early adulthood [19], and decline with age [20]. It is, therefore, important to explore the factors which affect the dental anxiety of both children and adolescents, including the influence of family milieu.

The present study aims to evaluate the influence of various family factors, including dental anxiety of both parents, on the dental anxiety of adolescents up to the age of 18 years. The null hypothesis (H_0_) was that no associations will be found between adolescents’ dental anxiety and none of the examined family unit variables (e.g., demographic variables, parent’s dental anxiety, and parent’s memories and feelings regarding previous dental treatment).

## 2. Materials and Methods

The study was conducted in full accordance with the World Medical Association Declaration of Helsinki. The Ethics Committee of Tel Aviv University approved all the study procedures. An informed consent form was signed by all parents.

The study included adolescents (ages 13–18 years) and their parents, who arrived for a routine check-up at a public dental clinic of a major dental corporate in the center of Israel.

In order to avoid bias due to previous multiple and/or traumatic dental treatments, adolescents who had experienced the following dental treatments were excluded: (i) previous treatment for emergency conditions; (ii) more than 5 dental restorations; (iii) a permanent tooth extraction; (iv) endodontic treatment; and (v) orthodontic treatment.

The study used questionnaires that were completed by adolescents and both of their parents (Appendix A).

The questionnaires were distributed during the first visit to the clinic. Adolescents were requested to complete their part on their own, without their parent/s being involved in the process. The accompanying parent was requested to complete the questionnaire in the clinic and have the other parent complete the questionnaire at home.

The questions included in the questionnaire (apart from DAS and demographic information, as described below) were agreed upon and tested for content validity by a group of subject matter experts (SMEs). The group consisted of three dentists and one clinical psychologist, who worked at the Tel Aviv University School of Dental Medicine and have extensive clinical and academic experience in working with patients suffering from dental anxiety. Each SME proposed questions for the study and the final questions were agreed upon as a consensus decision.

The following information was collected:

### 2.1. Information Collected from Adolescents

(a) General information—age, gender.
(b) Adolescent’s level of dental anxiety using the DAS questionnaire (A-DAS).
(c) Previous knowledge about dental treatment as received from parents:
  − Do you hear stories from your mother/father about their dental treatment (yes/no)?
  − If yes, are these stories nice/neutral/stressful or unpleasant?

### 2.2. Information Collected from Parents (Separate Questionnaire for Each Parent)

(a) Demographic and general information—age, gender, education, place of birth, marital status (married/separated/other), accompanied by (mother/father/both parents), number of children in the family, sequence of the treated adolescent among siblings (firstborn versus other), and time passed since the adolescent’s last dental visit (more or less than one year).
(b) Parent’s own level of dental anxiety using the DAS questionnaire—mothers’ DAS was marked as M-DAS and fathers’ DAS was marked as F-DAS.
(c) Each parent’s memories and feelings regarding previous dental treatment (Mem) are as follows:
  − Does the visit at the clinic arise previous memories from childhood regarding dental treatment (yes/no)?
  − If yes, do the memories trigger negative/positive/neutral feelings (Negative Mem, Positive Mem, Neutral Mem)?

### 2.3. Information Regarding Subjects’ Behavior at the Clinic

One of the researchers (MK) observed the adolescents and their parents as they entered the operatory room and recorded the following:

− Does the parent look tense/relaxed/detached (Parent tense, Parent relaxed, Parent detached)?
− Does the adolescent exhibit independent behavior (enters the operatory room on his/her own) or dependent behavior (seeks parent’s proximity, hides behind parent, refuses to sit by him/herself in the dental chair, etc.)?

Data analysis: Data were analyzed using SPSS software version 25 (Chicago, IL, USA) Differences between variables were assessed by *t*-test or one-way ANOVA. Pearson correlation coefficients were used to identify associations between the study variables. Stepwise regression analysis was used to determine the variables that best predict A-DAS. The significance level was set to 0.05.

## 3. Results

Questionnaires were distributed to 120 parents and their adolescent children. Although not an inclusion criterion, all the families consisted of married, heterogeneous couples.

Of the 120 families who were invited to take part in the study, 89% consented to participate. An additional 5% of the questionnaires were rejected due to improper completion. The final study population comprised 100 families (300 questionnaires), which represents an 84% response rate.

Demographic details of the participating families are presented in Table 1. The mean age of the treated adolescents was 14.87 ± 1.57 years (age range 13–18 years), with 48% girls. The majority (57%) had visited a dental clinic less than one year ago. The mean A-DAS score was 9.83 ± 2.05, with no significant differences between boys and girls (9.77 ± 1.94 and 9.89 ± 2.17, respectively).

There were significant differences between the M-DAS and F-DAS (10.51 ± 2.21 and 9.48 ± 2.38, respectively, *p* < 0.001), with mothers showing higher dental anxiety than fathers.

A moderate positive correlation was found between the dental anxiety of the adolescent and that of their parents (A-DAS and M-DAS, r = 0.79, *p* < 0.001; A-DAS and F-DAS, r = 0.52, *p* < 0.001). There was also a positive correlation between the dental anxiety of the two parents (M-DAS and F-DAS, r = 0.43, *p* < 0.001). Additional variables with a significant correlation to A-DAS were the number of children in the family (r = 0.369, *p* < 0.01) and parent’s education (r = −0.36 for father, r = −0.32 for mother, *p* < 0.01 for both).

Significant differences in A-DAS were evident in relation to several family variables (Table 2). Adolescents were more anxious when accompanied by their father, when their last dental visit occurred over one year ago, when not the firstborn child in the family, when the parent’s behavior at the clinic was defined as tense, and when parents’ (both mother’s and father’s) memories from their own previous dental encounters were negative.

In order to indicate which of the variables can serve as potential predictors of adolescents’ dental anxiety (A-DAS), a stepwise regression analysis was performed. Variables introduced in the equation were the variables listed in Table 2, in addition to M-DAS and F-DAS (Table 3).

The results indicate that variables best predicting an adolescent’s dental anxiety were the mother’s dental anxiety (M-DAS), the adolescent’s behavior in dental surgery (independent), being the firstborn child, the father’s dental anxiety (F-DAS), and the mother’s education.

## 4. Discussion

Dentists estimate that over a quarter of their adolescent/adult patients suffer from dental anxiety and report devoting about a quarter of their weekly work hours to treating such patients [21]. The toll of dental anxiety is high on both patients and dentists. While dentists feel committed to treating anxious patients, they also admit that treating uncooperative patients is stressful [21].

In recent years, a number of studies have attempted to evaluate the effect of vicarious learning (mainly through parents) on the development of dental anxiety in children [22,23,24,25]. Lara et al. [22] showed that among children aged 7–12 years, positive correlations exist between the parent’s and children’s dental fear scores and that the father’s dental fear is a mediating factor in the relationship between the mother’s and children’s fear scores. Olak et al. [23] confirmed this finding for children aged 8–10 years, and Coric et al. [24] reported similar findings for 7–15-year-old children.

In an interesting longitudinal study, Luoto et al. [25] followed children and their parents over 5 years, while the children grew from the age of 11–12 to 15–16 years. Their findings indicate that the prevalence of dental fear increases slightly over the years and seems to be more stable in adulthood than in childhood.

Undoubtedly, parents and family play a significant role in the development and persistence of dental anxiety among younger family members. Most of the studies relating to this issue considered relatively young children (mostly up to the age of 12 years) and referred to the influence of only one of the parents (either mother or father). Luoto et al. [25], criticized this approach, pointing out that both parents should be considered.

The present findings indicate that dental anxiety is higher in mothers than in fathers, which is in accordance with the results of previous studies showing that women are usually more dentally anxious than men [22,25,26]. However, the findings concerning the dental anxiety of adolescent girls versus boys are not so consistent. While Coric et al. [24], and Majstrovic [27] reported no gender-related differences in dental fear among young children, Lara et al. [22] concluded that schoolgirls are more dentally fearful than schoolboys and that children’s gender is a significant predictor of dental fear. In the present study, no such differences could be detected as far as adolescents are concerned.

In many cases, dental anxiety arises during childhood as a result of direct and/or vicarious learning. Environmental factors such as family may have a crucial effect on the development (and maintenance) of dental anxiety. Peretz et al. [10] showed that family factors, such as the number of children in a family, the parents’ age and education, or place of birth, influence dental anxiety levels among children between the ages of 6–14 years. In the present study, the mother’s level of education was one of the factors that played a significant role in the prediction of their adolescent child’s dental anxiety. Other factors included the dental anxiety levels of both parents, as well as being (or not being) the firstborn child in the family.

A number of studies have reported a significant correlation between the dental fear of parents and children, but the relationship varies according to the choice of parameters and the age of children [28]. In the present study, parents’ DAS scores correlated with one another, as well as with that of their adolescent child. This confirms the findings of Lara et al. [22] who demonstrated correlations between the levels of fear of the two parents, as well as between parents and their children. Similarly, Coric et al. [24] described the coexistence of dental fear in parents and their older children. Interestingly, the two groups differ in their opinion as to which parent’s dental anxiety predicts that of their child. While Coric et al. [24] concluded that only the mother’s dental anxiety is predictive of their children’s DAS score, Lara et al. [22] showed that when the levels of fear of both mother and father were jointly included in a regression model, only the fathers’ dental fear remained.

In the present study, the dental anxiety levels of both the mother and father (in addition to several other variables) were part of the final prediction model. Although the effect of the mother’s anxiety (M-DAS) in the model was stronger than that of the father’s (F-DAS), both variables were significant in predicting the child’s dental anxiety. Additional factors in the model were the adolescent’s behavior in the dental surgery, his/her order among siblings (firstborn versus not firstborn), and the mother’s level of education. The results of the model are in contrast to the report of Coric et al. [24], who reported that socioeconomic variables such as education do not correlate with children’s dental fear and anxiety. On the contrary, in the present study, the mother’s education was significantly predictive of the dental anxiety levels of the treated adolescents.

Several recent studies considered the child’s birth order in relation to their dental fear. Ghaderi et al. [29] reported that single children are less cooperative and more anxious, and that middle children are more cooperative than the other siblings. Aminabadi et al. [30] showed that firstborn children are at an increased risk of developing negative behavior and clinical and situational anxiety. The fact that both studies included relatively young children (5–7 years old) might have had a significant effect on the subjects’ behavior. In a study on older children (9–13 years old), Wu and Gao [31] showed that children with siblings tend to report higher levels of dental fear than only children. Their conclusion was that the family structure (nuclear or single-parent family), and the presence of siblings, are significant determinants of children’s dental fear. In the present study, all participating couples were married and heterogeneous. Only 6% of the participating families had a single child, and 42% of the participating adolescents were firstborns. Under these conditions, the firstborn participants manifested significantly lower dental fear scores than participants who came somewhere else in the family order of siblings.

Although not all of the studied variables were included in the final predictive model, some interesting results were detected. Adolescents’ dental anxiety was higher when their last dental visit was over 12 months ago, when the father was the accompanying parent, when the accompanying parent’s behavior was tense, and when one or both parents’ memories from their own previous experiences at the dental office were negative. This confirms the known fact that avoidance or postponing dental treatment among patients with dental fear is a common phenomenon [2,32]. Apparently, this starts already during adolescence.

It is important to note that negative memories from previous experiences at the dental office may act as an indirect, possibly even unconscious, way for parents to transfer dental anxiety to their children. Smith et al. [33] showed that parents relived their own negative experiences of dental treatment through words and expressions and possibly by delaying dental treatment for their children. This might also have been the case in the present study.

In a previous study carried out on adults, the best predictors of a decline in subjects’ dental anxiety over time were the evaluation of their past and present dentists and a secure pattern of attachment [18]. Although attachment patterns were not evaluated in the present study, the independent behavior of the adolescent patient while entering the dental surgery played a significant role in the predictive model. This suggests that patients exhibiting more independent behavior were not only less fearful but possibly also more secure in their relationship with the accompanying parent. Accumulating evidence from recent studies stresses the importance of attachment to parents in adolescence and emphasizes the role of the quality of attachment in the formation of an autonomic capacity to solve problems and cope with difficulties [34].

In contrast to the well-known myths concerning teenage rebellion against parental authority and influence, most adolescents are well-integrated into their families and have close relationships with significant others (parents) [35]. Both parents and siblings play an important role in the adolescents’ journey from childhood to adulthood and their relation to and coping with potentially stressful situations such as dental treatment. Parents’ fears and memories, even when subconscious and suppressed, can play a significant role in those of their children. An increased awareness of these issues can reduce the risk of the development of the dental-anxiety vicious cycle and increase children’s and adolescents’ ability for lifelong beneficial dental care. Paying closer attention to the relationships between the adolescent patient and his/her parents can increase the dentist’s understanding regarding the adequate treatment approach for the patient.

While investigating parents’ dental anxiety, memories, and feelings, the study did not investigate the parent’s feared specific dental procedures, such as local anesthesia. For example, many parents are concerned about the use of general anesthesia in pediatric dental treatment, a concern that can serve as a vicarious trigger for their children’s fear [36]. Moreover, while the use of computer-controlled local anesthetic delivery may lead to a reduction in pain related to anesthetic injection, and as a result, to a reduction in anxiety [37], in the present study, local anesthesia was administered in the traditional way. Further studies, including more detailed information on the family milieu (e.g., including information about parents’ and siblings’ specific fears, attachment patterns, etc.) should be carried out to enable a better understanding of the influence of family milieu on children’s and adolescents’ dental anxiety.

## 5. Conclusions

Parents’ dental anxiety, the mother’s level of education, the adolescent’s behavior at the dental office, and being the firstborn child in the family have shown a significant ability to predict adolescents’ dental anxiety. The results showed that intra-family relationships play an important role in defining the level of dental anxiety in adolescent patients.

## Figures and Tables

**Table 1:** Family demographic data.

		%	Mean ± SD
Mother: Place of Birth	Israel	67	
	Former Soviet Union	30	
Education (years)			12.97 ± 2.58
Age			40.67 ± 4.55
Father: Place of birth	Israel	70	
	Former Soviet Union	25	
Education (years)			13.73 ± 2.94
Age			43.93 ± 5.10
Family: No. of children in family	One	6	
	Two	31	
	Three	37	
	Four and more	26	
Birth order of the treated adolescent	Firstborn	42	
	Second	44	
	Third or above	14	

**Table 2:** Adolescent’s dental anxiety (A-DAS) according to family variables.

Family Variables:	A-DAS
Accompanied by: Father (*n* = 33)	1.75 ± 11.10
Mother (*n* = 32)	9.95 ± 1.45
Both parents (*n* = 35)	8.50 ± 1.95
*p* (ANOVA)	<0.01
Last dental visit: Less than 1 year ago (*n* = 57)	9.25 ± 2.10
Over 1 year ago (*n* = 43)	10.70 ± 1.60
*p* (*t*-test)	<0.01
Birth Order: Firstborn (*n* = 42)	9.20 ± 2.2
Not firstborn (*n* = 58)	10.30 ± 1.8
*p* (*t*-test)	<0.01
Parent’s behavior: Tense (*n* = 19)	10.95 ± 1.45
Relaxed (*n* = 81)	9.65 ± 2.10
*p* (*t*-test)	<0.05
Adolescent: Dependent (*n* = 26)	11.55 ± 1.40
Independent (*n* = 74)	9.25 ± 1.90
*p* (*t*-test)	<0.01
Mother: Negative Mem. (*n* = 55)	10.80 ± 1.75
Positive Mem. (*n* = 8)	6.90 ± 1.55
Neutral Mem. (*n* = 37)	9.55 ± 1.50
*p* (ANOVA)	<0.01
Father: Negative Mem. (*n* = 27)	12.00 ± 2.0
Positive Mem. (*n* = 7)	6.70 ± 1.60
Neutral Mem. (*n* = 66)	8.70 ± 1.60
*p* (ANOVA)	<0.01

In parenthesis—number of subjects.

**Table 3:** Variables predicting adolescent’s dental anxiety.

Model	Unstandardized Coefficients		Sig.	95.0% Confidence Interval for B	
	B	Std. Error		Lower	Upper
(Constant)	3.803	0.917	0.000	1.981	5.624
M-DAS	0.573	0.058	0.000	0.458	0.689
Adolescent’s independent behavior.	0.873	0.275	0.002	0.328	1.418
Firstborn	−0.449	0.232	0.056	−0.909	0.011
F-DAS	0.133	0.054	0.015	0.026	0.240
Mother’s education	−0.100	0.044	0.027	−0.188	−0.012

## Data Availability

The data presented in this study are available on reasonable request from the corresponding author.

## Appendix A. Questionnaire

***Questions to Parent***	***Answers***
1. Who is the parent that usually accompany the child?	Mother Father Both
2. Age	
3. Gender	Female Male
4. Place of Birth	Israel Former Soviet Union Other
5. Marital status	Married Separated Other
6. Education (in years)	
7. Number of children in the family	
8. The children under our treatment is	The firstborn; the second; the third or above
9. How much time has passed since the adolescent’s last dental visit?	More than one year Less than one year
10. Does the visit at the clinic arouse previous memories from childhood regarding dental treatment?	Yes No
11. If yes, do the memories trigger negative/positive/neutral feelings?	Negative feelings Positive feelings Neutral feelings
12. DAS questionnaire	Four questions related to dental anxiety
***Questions to Adolescent***	
1. Age	
2. Gender	Girl Boy
3. Do you hear stories from your mother/father about their dental treatment?	Yes No
4. If yes, are these stories nice/neutral/stressful or unpleasant?	Nice Neutral Stressful or unpleasant
5. DAS questionnaire	Four questions related to dental anxiety
***Subjects’ behavior at the clinic (filled by one of the researchers)***	
1. How does the parent look when entering the operatory room?	Tense Relaxed Detached
2. Does the adolescent exhibit independent behavior or a dependant behavior?	Independent behavior = enters the operatory room on his/her own. Dependant behavior = seeks parent’s proximity, hides behind parent, refuses to sit by him/herself in the dental chair, etc.).
